# Apple *B-box* factors regulate light-responsive anthocyanin biosynthesis genes

**DOI:** 10.1038/s41598-019-54166-2

**Published:** 2019-11-28

**Authors:** Blue J. Plunkett, Rebecca Henry-Kirk, Adam Friend, Robert Diack, Susanne Helbig, Katriina Mouhu, Sumathi Tomes, Andrew P. Dare, Richard V. Espley, Joanna Putterill, Andrew C. Allan

**Affiliations:** 1 The New Zealand Institute for Plant and Food Research Limited (PFR), Mt Albert, Private Bag 92169, Auckland, New Zealand; 2 PFR, 55 Old Mill Road, RD 3, Motueka, 7198 New Zealand; 3 BIOTECON Diagnostics GmbH, Hermannswerder 17, 14473 Potsdam, Germany; 4 Faculty of Agriculture and Forestry, University of Helsinki, Helsinki, Finland; 5 School of Biological Sciences, University of Auckland, Private Bag 92019, Auckland, New Zealand

**Keywords:** Light responses, Secondary metabolism

## Abstract

Environmentally-responsive genes can affect fruit red colour via the activation of MYB transcription factors. The apple *B-box (BBX)* gene, *BBX33/CONSTANS-like 11 (COL11)* has been reported to influence apple red-skin colour in a light- and temperature-dependent manner. To further understand the role of apple *BBX* genes, other members of the *BBX* family were examined for effects on colour regulation. Expression of 23 *BBX* genes in apple skin was analysed during fruit development. We investigated the diurnal rhythm of expression of the *BBX* genes, the anthocyanin biosynthetic genes and a MYB activator, MYB10. Transactivation assays on the *MYB10* promoter, showed that BBX proteins 1, 17, 15, 35, 51, and 54 were able to directly function as activators. Using truncated versions of the *MYB10* promoter, a key region was identified for activation by BBX1. BBX1 enhanced the activation of MYB10 and MdbHLH3 on the promoter of the anthocyanin biosynthetic gene *DFR*. In transformed apple lines, over-expression of BBX1 reduced internal ethylene content and altered both cyanidin concentration and associated gene expression. We propose that, along with environmental signals, the control of *MYB10* expression by *BBX*s in ‘Royal Gala’ fruit involves the integration of the expression of multiple *BBX*s to regulate fruit colour.

## Introduction

Apple is an important fruit crop, both commercially and from a dietary nutrition perspective. Compounds found in apple have been shown to have positive effects on human health^1,2^. These compounds are derived from secondary metabolites and comprise a diverse set of bioactive phytochemicals including phenylpropanoids, which contain the flavonoid subclass that includes anthocyanins, the pigments responsible for the red colour of apple. Potential health benefits include anti-inflammatory, anti-carcinogenic, and antioxidant properties^3,4^. Fruit colour is also a strong market driver, with consumer preferences varying between countries and regions^5,6^. In general, consumer appeal tends towards red colour, with red apples being preferred to yellow or green cultivars^5–7^. Apple colour and patterning varies naturally within and between cultivars, and in many apple cultivars this is influenced by environmental conditions, modulating the concentrations of chlorophyll, carotenoid, and anthocyanin^8,9^. The red colour of apple skin is composed almost exclusively of the anthocyanin derivative, cyanidin-3-*O-*galactoside.

Anthocyanin is important for plants as it protects against photo-oxidative and heat damage caused by sunlight, as well as influencing pollination and seed dispersal^10–12^. The accumulation of anthocyanins in plants appears to be linked to biotic and abiotic stress, with anthocyanin concentrations being increased in response to herbivory, fungal and viral pathogens, wounding, temperature, high light and UV, mineral imbalance, drought, salinity, anoxia, ozone, herbicides^13^. Research on the regulation of anthocyanin production has identified both the biosynthetic pathways genes^14–16^ and the major regulating transcription factors (TFs), comprising the MYB-bHLH-WD40 (MBW) complex^17^. This complex binds promoter regions of anthocyanin biosynthetic genes and *MYB10* and increases transcription, as shown for Arabidopsis (*Arabidopsis thaliana*)^17^, nectarine^18^, petunia (*Petunia hybrida*)^15^, strawberry^19^, legumes^20^, Norway spruce^21^, and apple^22^, and reviewed by Li^23^.

The MYBs that regulate anthocyanin accumulation in apple have been well studied^22,24–26^. Alleles *MYBA* and *MYB1* are identical and share 98% sequence homology with *MYB10*, which differs by three amino acids in the open reading frame^25^. The R2R3 binding region of these and anthocyanin-related MYBs in other plant species is highly conserved^25^. In apple, MYB10, bHLH3/33 and a WD40 protein TTG1 have been shown to form the MBW complex seen in other species to regulate anthocyanin concentrations^26^. Patterning of apple fruit skin includes striped and blushed, and this variation can be explained by the differential expression of *MYB10* and degree of methylation in the promoter^27^.

Repressors also play an important role in the regulation of anthocyanin production. The genes involved and the mechanisms by which they operate are beginning to be resolved. For example, two repressors have been reported in petunia, MYB27 and MYBx^15,28^. MYB27 is a R2R3 type MYB, which can interfere with the MBW complex by preventing its formation or being incorporated, and converting the complex into a repression complex. MYBx is a R3 type MYB that also appears to compete for interaction with the bHLH component of the MBW complex. MYB27 contains an EAR (ethylene-responsive element binding factor associated amphiphilic repression domain) and TLLLFR motif, both of which appear to confer the ability to repress anthocyanin^15^. MYBL2, an R3-MYB, has been identified in Arabidopsis as a repressor of anthocyanin and also contains the TLLLFR motif^29,30^. Similar repression mechanisms have also been identified in strawberry^31^. In apple, a family of MYB repressors of anthocyanin were identified after heat treatment and were characterised by the EAR motif^32^.

Repression and activation of the anthocyanin pathway is responsive to environmental signals. Lin-Wang *et al*.^32^ have shown that heat reduces apple skin *MYB10* expression, while Xie *et al*.^33^ showed that cold induces bHLH3 to bind with MYB1 and upregulate the promoters of anthocyanin biosynthetic genes. Appropriate light conditions are also required for anthocyanin accumulation in many apple cultivars^34,35^ and anthocyanin has been shown to increase in response to ultraviolet-B (UV-B) irradiation and to absorb UV-B^7,36^. Long-term changes, enduring after six weeks of postharvest storage, were found in apples grown on trees covered by a canopy of UV-reducing film^37^. The reduction of UV affected a wide range of fruit qualities, including a delay in ripening, reduction in fruit size, and decrease of anthocyanin and flavonol concentrations. Many of the transcription factors responsible for these environmentally induced anthocyanin changes have been identified. As a consequence of these genes being responsive to environmental signals they can exhibit expression rhythms that follow the stimulus. These rhythms can be detected by measuring transcription. In Arabidopsis, ELONGATED HYPOCOTYL 5 (HY5), a basic leucine zipper (bZIP) transcription factor, receives signals from photoreceptors and regulates anthocyanin by directly binding promotors of *PAP1* (*AtMYB75*) and anthocyanin biosynthetic genes^38^. HY5 is also involved in low temperature-induced anthocyanin accumulation^39^. LIGHT-REGULATED ZINC FINGER PROTEIN 1 (LZF1, or B-BOX DOMAIN PROTEIN 22) has been found to act downstream of HY5 to inhibit anthocyanin^40^. The photoreceptor UV RESISTANCE LOCUS 8 (UVR8) provides light-signalling control of various pathways, including regulating *HY5* and interacting with CONSTITUTIVE PHOTOMORPHOGENIC 1 (COP1)^41,42^. COP1 is a RING finger type ubiquitin E3 ligase involved in the targeted degradation of various anthocyanin influencing factors, including MYB1 in apple^43–45^.

Although many transcription factors have been identified in the regulation of anthocyanin biosynthesis, a full understanding of how light regulates this process is still being developed. The role *BBXs* play is an example. Studies focusing on specific members of the BBX gene family in apple have shown that UV-B light in particular upregulates *MdCOL11/BBX33* expression in a temperature-dependent manner^7^. *MdBBX33* is a close homolog of *AtBBX22/LZF1*, also known as *STH3* (Salt Tolerance Homolog 3) and *DBB3* (Double B-Box zinc finger 3)^7,40,46^. Bai *et al*.^7^ generated transgenic lines of Arabidopsis overexpressing apple *BBX33*, which had increased anthocyanin accumulation in seedlings and showed also that the expression profiles of *BBX33* and *MYB10* are related during different temperature and light regimes. Using dual luciferase assays it was shown that BBX33 upregulates the *MYB10* promoter. These findings suggest that BBX33 functions downstream of HY5 and upstream of MYB10 as a component in the environmental sensing pathway, which leads to anthocyanin production in apple.

Discovery of the first *BBX*, in Arabidopsis, came from characterising the flowering pathway. CONSTANS (CO/BBX1) was shown to be important for regulating transcription levels of floral integrators where it is a key factor controlling photoperiodic flowering response in Arabidopsis^47–51^. CO/BBX1 was subsequently identified as a member of the larger *BBX* gene family. This is a family characterised by the proteins they encode possessing two highly conserved domains, an N-terminal zinc finger BBX^52,53^ and a C-terminal CCT (CO, CO-like, TIMING OF CAB EXPPRESSION 1) domain^54–56^. Both domains are involved with mediating transcriptional regulation and protein-protein interaction. Arabidopsis has 32 *BBX* genes (1–32), which fall into five groups (I-V) based on common variations in the conserved BBX and CCT domains^53^. Twenty-one of the 32 *AtBBX* gene sequences contain two BBXs in tandem, the remaining 11 contain one BBX^55,57^. This classification of *BBX* genes using number of conserved motifs has been reiterated by Huang *et al*.^58^ in rice. Further analysis, which included comparison of BBX sequences across a diverse range of species, has been conducted by Crocco and Botto^53^ where 214 BBX proteins were identified and compared, providing evolutionary insight into these genes.

More recently, Liu *et al*.^59^ reported a comprehensive list of the apple *BBX* gene family. A total of 64 *MdBBX*s were identified. The phylogeny and motif structure were reported, along with chromosomal locations. Although naming these genes as ‘*COL*s’ (for *CONSTANS-LIKE*) has been reported^56,60–64^, Liu *et al*.^59^ and others^53,55^ selected ‘*BBX’* as the convention so as to include genes that do not contain the 3’ CCT motif, and to align with the naming convention reported by Khanna *et al*.^57^ in Arabidopsis.

Here we examined members of the *BBX* gene family in apple, using the naming convention from Liu *et al*.^59^, with an emphasis on potential roles in anthocyanin regulation. The expression of 23 *BBX*s, representing the five sub-clades in the gene family, in *Malus* x *domestica* ‘Royal Gala’ fruit skin during fruit development was assessed. Given that many *BBX* genes, including Arabidopsis *CO*, *COL1* and *COL2*, show diurnal patterns of expression^64,65^, a detailed examination of diurnal expression patterns in both candidate apple BBX genes and anthocyanin structural genes was performed. Expression profiles of anthocyanin biosynthetic genes and the MYB regulator were tested for expression correlations. A subset of the BBX proteins were screened for their ability to activate the promoter of *MYB1/10*, which was also tested for any dependent BBX-related motifs. Furthermore, the activation of the promoter of the anthocyanin biosynthetic gene *DIHYDROFLAVONOL 4-REDUCASE* (*DFR)* by MYB10 and bHLH3 was assessed in the presence of BBX1. Finally, we generated and characterized five transgenic lines of apple trees overexpressing *BBX1* and measured postharvest metrics and metabolite content differences compared to wildtype.

## Materials and Methods

### Plant material

Apple fruit tissue used for the development series was sampled from ‘Royal Gala’ trees grown in standard conditions in a glasshouse in 2012/2013 at seven time points during development: 35, 65, 85, 110, 120, 130 and 140 days after full bloom (DAFB) at 1300 h. Full bloom occurred on 28 September 2012 and at each time point five fruit were harvested and pooled. Apple fruit tissue used for diurnal sampling were collected from ‘Royal Gala’ apple trees in the Plant and Food Research orchard, Motueka, Nelson, New Zealand, on 8–10 February 2011 (season one) and from the Plant and Food Research orchard, Mt Albert, Auckland, New Zealand, on 27 February - 2 March 2012 (season two). Apple leaf tissue was harvested at the same time as fruit using five pooled leaves per time point. Fruit sampling began at 120 DAFB. Due to length of time course, number of fruit harvested per time point had to be limited to three fruit at each time point, which occurred at 1300 h, and then pooled. To help with this, two seasons were sampled. Apple trees over-expressing *BBX1* were grown in the Plant and Food Research glasshouse in Auckland. Three pooled fruit were used for each replicate and run in quadruplicate for gene expression analysis and the average reading for five fruit is presented for ethylene, weight, firmness, and soluble solids content (measured as °Brix). High performance liquid chromatography (HPLC) and liquid chromatography-mass spectrometry (LCMS) data are also presented as the average reading from five fruit. For each fruit all of the skin was removed using a scalpel and immediately frozen in liquid nitrogen along with leaf samples, ground to powder without thawing, then stored at −80 °C until RNA extraction for RT-qPCR analysis.

### Promoter and gene cloning

Full-length coding sequences of *MYB10*, *MYB8*, *bHLH3*, *bHLH*15 and *BBX1*, *9*, *10*, *15*, *17*, *33*, 35, *37*, *42*, *43/44*, *47*, 51, *54*, *57* were isolated from ‘Royal Gala’ cDNA and inserted into the pGEM-T Easy vector (Promega, Madison, USA) before being inserted into the pHEX2 binary vector via restriction site cloning^66^. Promoter sequences for the R_1_ allele of *MYB10*, *DFR*, *BBX1*, and *BBX15* were isolated from ‘Royal Gala’ genomic DNA and inserted into the cloning site of pGreenII08005’-LUC as previously described by Espley *et al*.^67^. Truncated versions of the promotor of *MYB10* (∆a and ∆b) were generated by designing primers to amplify 834 bp and 405 bp fragments upstream of the *MYB10* start site. Primer sequences are given in Supplementary Table 1.

### Dual luciferase assay of transiently transformed *Nicotiana benthamiana* leaves

*Nicotiana benthamiana* plants were used for transient transformation assays. These plants were grown at Plant and Food Research, Auckland, according to standard glasshouse management practices. *Agrobacterium tumefaciens* GV3101 was transformed by electroporation to introduce constructs. *N*. *benthamiana* leaves were transiently transformed for the dual luciferase assay as previously reported by Lin-Wang *et al*.^68^.

### Metabolite analysis

Anthocyanin content was measured by HPLC. For the more comprehensive analysis of the *BBX1* over-expressing lines, HPLC was used to detect anthocyanins and polyphenols. Ground apple skin tissue (100 mg) was freeze-dried for 12 hours. Acidified (0.5% HCL) methanol (5x volume) was added to the tissue and placed in the dark for 3 hours to extract. Samples were centrifuged at 16,000 rpm for 4 minutes, then supernatant transferred to a new tube for vacuum evaporation. Extract was then re-suspended in 500 µL of 20% methanol, filtered into a new tube and vacuum evaporated. Extract was then finally re-suspended in 20 µL methanol for analysis. Extract was analysed on a Dionex Ultimate 3000 RS system as previously described by Dare *et al*.^69^.

### Gene expression analysis by RT-qPCR

RNA was isolated using Spectrum™ RNA extraction kit (Sigma-Aldrich) according to the manufacturer’s instructions. RNA quantification was performed using a NanoDrop™ ND-1000 UV-Vis Spectrophotometer (NanoDrop Technologies, Thermo Scientific, USA) and visualized by gel electrophoresis. First strand cDNA synthesis of 2 µg total RNA was performed using Qiagen QuantiTect® Reverse Transcription Kit (Qiagen). Expression analysis was performed using four technical replicates on a Roche LightCycler® 480 platform and LightCycler FastStart SYBR Green I Master Mix as described previously^22^. Data was analysed using the Target/Reference ratio calculated with the LightCycler 480® software version 1.5.0.39 (Roche) and primer efficiency corrections added. CP values for housekeeper, *Actin*, did not vary by more the 1.72 cycles during the 48 h and 52 h of sampling for season one and two respectively. Primers were designed to optimise efficiency as previously described^32^. Primer sequences are given in Supplementary Table 1.

### Internal ethylene assessment

Internal ethylene concentration of apple fruit was determined using a needle and syringe to extract gas from the internal core cavity of attached fruit during season 2 of sampling at 130 DAFB at 1300 h. *BBX1* over-expressing lines samples were taken at 125 DAFB at 1300 h. Core cavity gas (1 mL) was injected into a gas chromatograph (Hewlett Packard, 5890 series II) and run according to the method of Johnston *et al*.^70^.

### Statistical analysis

To test correlations between genes, the gene expression data were used to generate correlation coefficient values using two-sided Pearson’s product-moment correlation in R version 3.2.4^71^. Differences between averages were analysed using Analysis of Variance (ANOVA) in GenStat version 17.1.0.14713. *Post hoc* pairwise differences were determined using Fisher’s LSD test at the 5% significance level^72^.

### Stable transformation of apple

The cDNA sequence of apple *BBX1* was cloned into the binary vector pSAK277^22^ driven by the 35 S promoter and transferred into *Agrobacterium* strain LBA4404. Transgenic lines of ‘Royal Gala’ plants were generated by *Agrobacterium*-mediated transformation of leaf pieces using a method previously reported by Yao *et al*.^73^. Transgenic plants were rooted and grown in a containment glasshouse at The New Zealand Institute for Plant and Food Research Limited (PFR), Auckland, New Zealand, with flowering and fruiting occurring three years after planting.

## Results

### Anthocyanin concentration and gene expression of the *BBX* family in fruit skin during development

**Figure 1:**
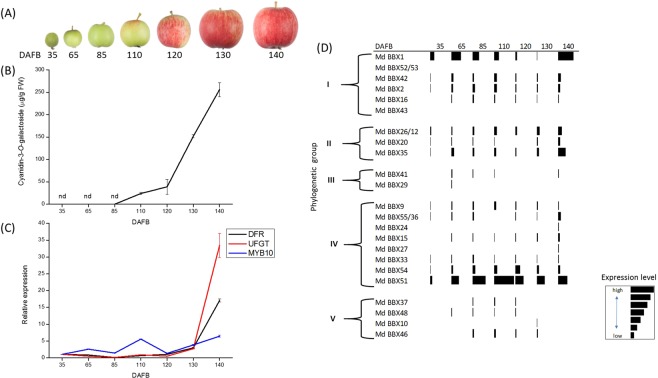
Assessment of skin anthocyanin accumulation during apple fruit development compared with *BBX* gene expression. Apple fruit were assessed at seven time points; 35, 65, 85, 110, 120, 130, and 140 DAFB. (**A**) Photographs of representative apple fruit at each stage of development assessed showing accumulation of red colour. (**B**) Cyanidin-3-*O-*galactoside accumulation in apple fruit skin at seven time points during development. Values are the average concentration of cyanidin as determined by HPLC from two pooled fruit per time point. Time points where no cyanidin was detected are indicated by ‘nd’. Error bars show SE of four technical replicates. (**C**) Relative expression of *DFR*, *UFGT*, and *MYB10* in apple fruit skin. Expression is relative to *Actin* normalized to 35 DAFB. Error bars show SE of four technical replicates. (**D**) Table showing relative expression level of selected *BBXs* in apple fruit skin at seven time points during development. Phylogenetic groups reported by Liu *et al*.^59^ indicated. Black bar at each DAFB indicates level of expression relative to the other *BBX* genes. Key shows that the length of the black bars indicates level of *BBX* expression relative to *Actin* and normalised to highest expressed *BBX* (BBX51 at 110 DAFB).

Apple fruit were assessed at seven time points during development: 35, 65, 85, 110, 120, 130, and 140 DAFB (Fig. 1A). At each time point, concentrations of cyanidin-3-*O*-galactoside, the expression of apple anthocyanin biosynthetic genes *DFR* and *URIDINE DIPHOSPHATE (UDP)-GLUCOSE FLAVONOID 3-O-GLYCOSYLTRANSFERASE* (*UFGT*), and the expression of anthocyanin-regulating transcription factor *MYB10*, as well as that of 23 genes from the *BBX* family were measured. Cyanidin-3-*O*-galactoside content was detectable at 110 DAFB, increasing up to 140 DAFB (Fig. 1B). Expression of *DFR*, *UFGT*, and *MYB10* increased during development, with the highest expression for all three genes seen at 140 DAFB (Fig. 1C). Transcripts of a subset of the *BBX* family genes, representing all five sub clades^59^, were also examined in apple skin during fruit development (Fig. 1D). This identified *BBX*s with expression patterns that coincided with anthocyanin accumulation in developing fruit. The highest and lowest expression for the genes varied over the six time points measured within and between the different groups of *BBX*s. Transcript abundance of many of the *BBX*s started low and then increased during fruit development (data presented at higher resolution in Supplementary Fig. 1). *BBX51* and *BBX1* were the two most highly expressed *BBX*s examined. Expression of *BBX51* was highest at 110 DAFB, while *BBX1* had an expression peak early in development (65 DAFB) and then again at 140 DAFB. Some genes, such as *BBX15*, were relatively lowly expressed but still displayed increasing expression during fruit development (Supplementary Fig. 1). Overall, *BBX*s from groups I, II and III had variable patterns of gene expression in the skin during fruit development, with the highest expression occurring early, middle, or late in the sampling. Group IV *BBX*s generally displayed their lowest expression at 35 DAFB, with the exception of *BBX27* (Supplementary Fig. 1). Most group V *BBX*s started with low expression and peaked at different times around the mid-range of the sampling period.

### Measuring diurnal patterns of transcription in apple leaves and fruit

**Figure 2:**
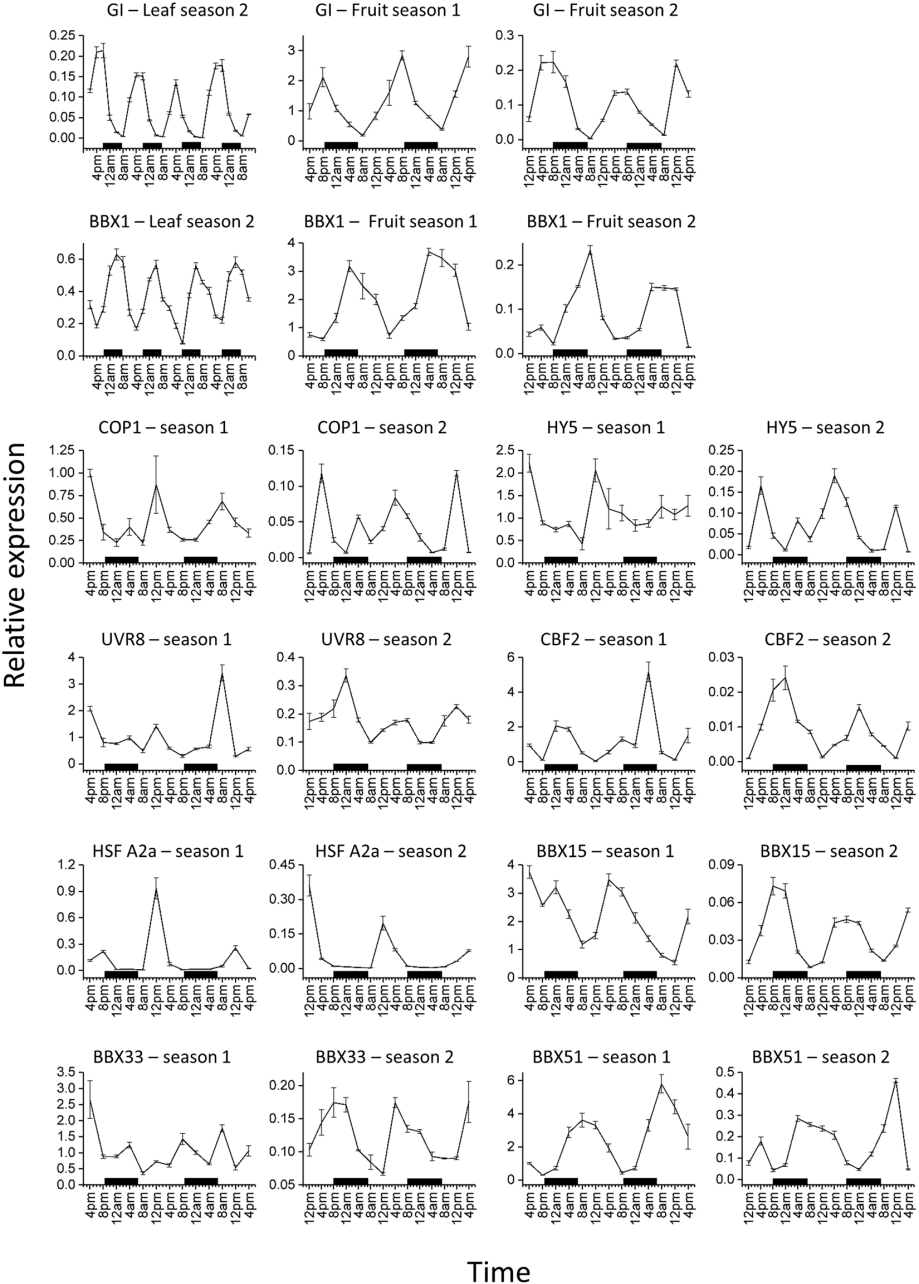
Diurnal gene expression levels at four hour intervals over two seasons. Gene expression for apple *Gigantea (GI)* and *BBX1* in apple leaf tissue and fruit skin tissue labelled, all other genes are fruit skin results. Other genes tested were light-associated *COP1*, *HY5*, and UVR8, temperature-linked genes *CBF2* and *HSFA2a* and BBX genes 15, 33, and 51 from BBX group IV over two seasons. Expression results were normalised to *Actin* and error bars are SE of four technical replicates. Daylight hours were from 0640 h to 2040 h for season 1 and from 0700 h to 2000 h for season 2, rounded to the nearest 10 min. Black bars indicate night.

Having demonstrated that the transcription of certain *BBX* genes increases during fruit development in a similar pattern to the concentration of anthocyanin and key anthocyanin-related genes, we then tested the transcript abundance of selected genes during a diurnal time series at 120 DAFB. This is the stage at which anthocyanin is rapidly accumulating. To confirm that variation in expression could be detected in apple diurnally, we tested transcription of apple *GIGANTEA* (*GI*) in leaf and fruit tissue (Fig. 2). *GI* was selected as a positive control because of its role in the circadian pathway and its known pattern of expression^74,75^. *GI* was found to be expressed in a highly regular pattern (high in the evening and low in the morning) in accordance with findings in Arabidopsis^74,75^. The expression of *BBX1* showed a distinct pattern of expression in the apple leaf, with the highest expression levels around 0400 h and lowest in the evening. Other genes, such as *BBX51*, *DFR*, *HY5*, and *COP1* also exhibited daily transcript fluctuations in apple leaves (Supplementary Fig. 2).

Diurnal rhythms of gene expression were assessed in fruit skin over two seasons and two locations, to control for environmental and seasonal variation (Fig. 2). *GI* expression in skin showed a diurnal expression rhythm with the highest expression in the evening at 2000 h and lowest at 0800 h for both seasons and sites, as was seen in leaves. *BBX1* peaked at 0400 h and was lowest in the evening, as was seen in leaves. A selection of light and temperature genes were also measured in apple skin to ascertain if detection of daily rhythms for genes reported to be involved in light and temperature perception was also possible in apple skin. These included *COP1*, *HY5*, and *UVR8* involved in light response (Fig. 2), and *CBF2* and *HSFA2a* involved in temperature-regulated expression (Fig. 2)^76,77^. A further six genes were tested (Supplementary Fig. 3). *COP1* and *HY5* expression patterns were similar, displaying peaks during daylight. These findings for apple *COP1* support work in Arabidopsis where *COP1* expression is high in the afternoon^43^. Peaks in the expression of *UVR8* coincided with light periods except for the first night of season 2. As expected, *CBF2* expression was elevated at night and *HSFA2a* was highest during the day (Fig. 2).

*BBX*s 51, 33, and 15 were also selected for assessment (Fig. 2). *BBX51* was chosen as it showed the highest expression during development (Fig. 1D), *BBX33* because of its involvement in anthocyanin accumulation^7^, and *BBX15* as it showed increased transcription over fruit development (Fig. 1D). Expression of *BBX51* was highest in the morning and lowest in the evening. Expression of *BBX33* was not found to be as rhythmic as the other *BBX* genes tested for season 1, and in season 2 it showed highest expression in the evening and lowest in the morning. *BBX15* was also highest in the evening and lowest in the morning, for both seasons tested.

### Correlation analysis of *MYB10* and anthocyanin pathway genes

**Figure 3:**
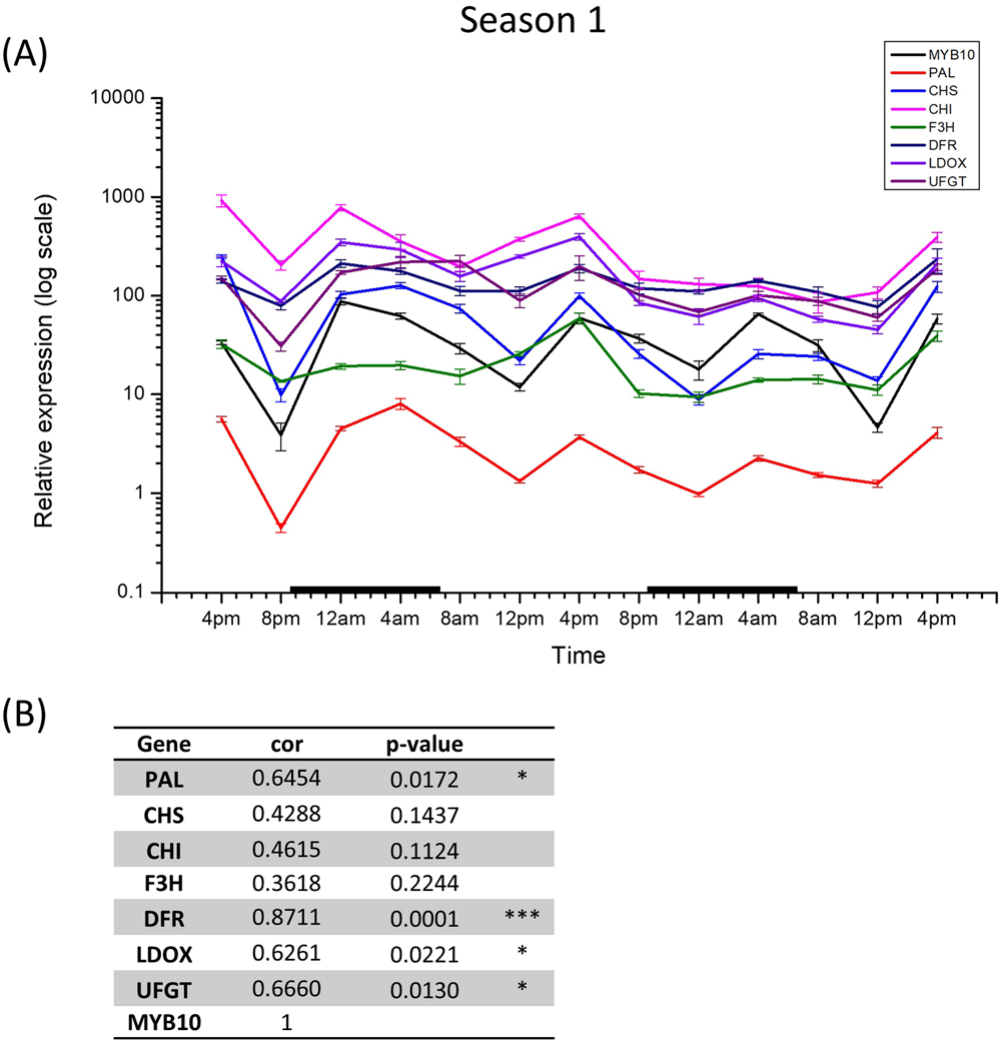
(**A**) Diurnal gene expression at four-hour intervals for *MYB10* and seven anthocyanin biosynthetic genes (*PAL*, *CHS*, *CHI*, *F3H*, *DFR*, *LDOX*, and *UFGT*). Expression was normalised to *Actin* and error bars are SE of four technical replicates. Daylight hours were 0640 h to 2040 h, rounded to the nearest 10 min. Black bars indicate night. (**B**) Table of Pearson’s product-moment correlation coefficient values for seven anthocyanin biosynthetic genes compared to *MYB10* (*p*-value < 0.05*, <0.01**, <0.001***).

To further investigate diurnal gene expression in apple fruit skin tissue, we analysed the expression of anthocyanin structural genes *PHENYLALANINE AMMONIA LYASE (PAL)*, *CHALCONE SYNTHASE (CHS)*, *CHALCONE ISOMERASE (CHI)*, *FLAVANONE 3-HYDROXYLASE (F3H)*, *DFR*, *LEUCOANTHOCYANIDIN DIOXYGENASE/ANTHOCYANIDIN SYNTHASE (LDOX/ANS)*, *UFGT*, and the *MYB10* TF (Fig. 3A). All the biosynthetic genes have a similar diurnal expression pattern to *MYB10*, with correlation analysis confirming a positive linear relationship, with four of the seven genes tested being statistically significant (*p* < 0.05) (Fig. 3B). However, no direct diurnal correlation with *MYB10* and *BBX* transcripts was found (Supplementary Table 2), even when taking into account a possible delay in the correlation by analysing for the effect on *MYB10* with a four- or eight-hour delay (data not shown).

### Activation of the MYB10 promoter by *BBX* genes

**Figure 4:**
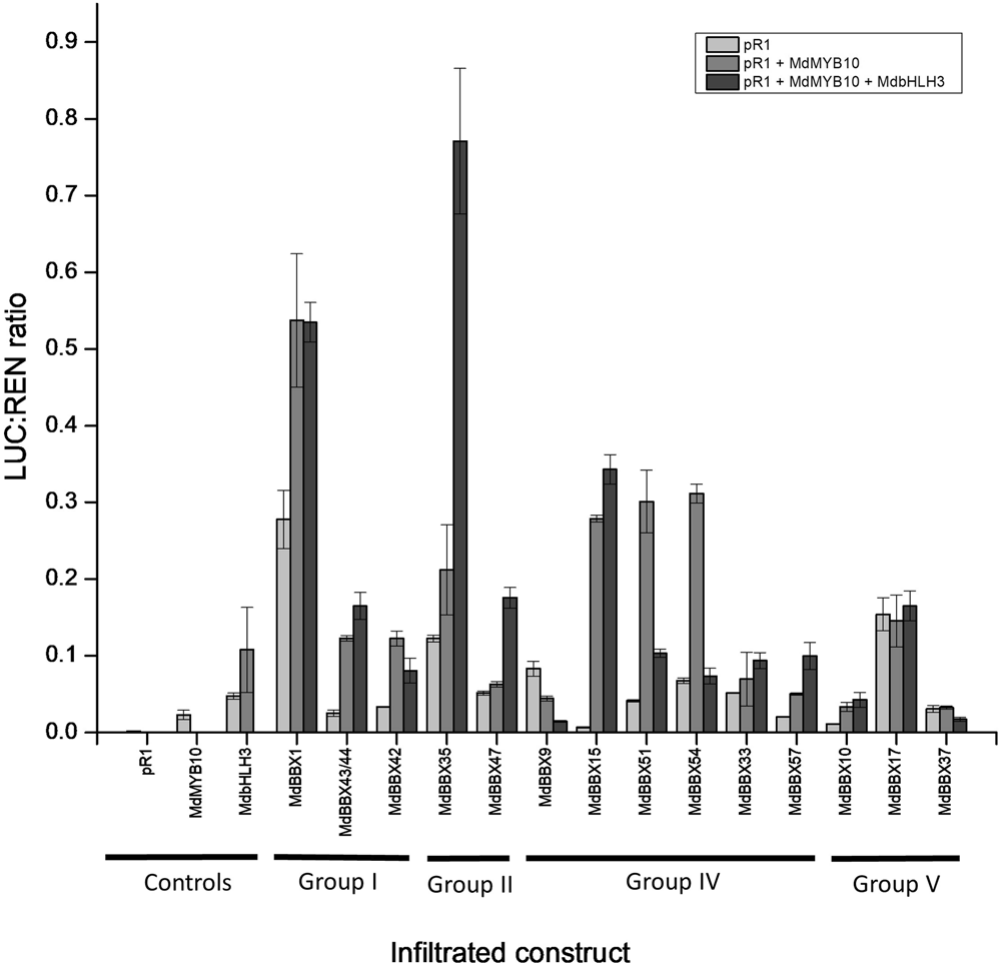
Transient expression assay of apple BBXs effect on the promoter of apple *MYB10* in tobacco. pR_1_ denotes the MYB10 promoter to distinguish it from the MYB10 protein. Each BBX was infiltrated with pR1 (light grey bars), with pR1 and MYB10 (medium grey), and with pR1, MYB10, and bHLH3 (dark grey). Phylogenetic groups as reported by Liu, *et al*.^59^ are indicated (groups I, II, IV, and V) and error bars show SE of four biological replicates.

Members of the apple *BBX* gene family were selected for promoter activation assays based on their expression during development (Fig. 1D and Supplementary Fig. 1). These 14 *BBX* genes were screened for activation of the *MYB10* promoter using the dual luciferase system in *N*. *benthamiana*. The *MYB10* promoter was infiltrated alone, or co-infiltrated with MYB10 + /- MdbHLH3 (Fig. 4). Compared with the controls, *BBX1* alone activated the *MYB10* promoter and showed an additive affect when co-infiltrated with *MYB10*, but no further activation was found when *bHLH3* was added. *BBX51* and *BBX54* both activated the promoter when co-infiltrated with *MYB10*, but this effect was largely lost after the addition of *bHLH3*. *BBX15* did not activate the promoter alone; however, the promoter was activated by the addition of *MYB10* and *MYB10* plus *bHLH3*. The highest activation of the promotor was when *BBX35* was co-infiltrated with *MYB10* and *bHLH3*. From these data, we identified *BBX1*, *15*, *35*, *51* and 54 as *BBX*s capable of activating the promoter of *MYB10*. Furthermore, the addition of the *bHLH* co-factor gene displayed all three possible effects, depending on the *BBX*: no change, or decreased or increased activation.

### Deletions in the *MYB10* promotor reveal specific sites required for BBX1 activation

**Figure 5:**
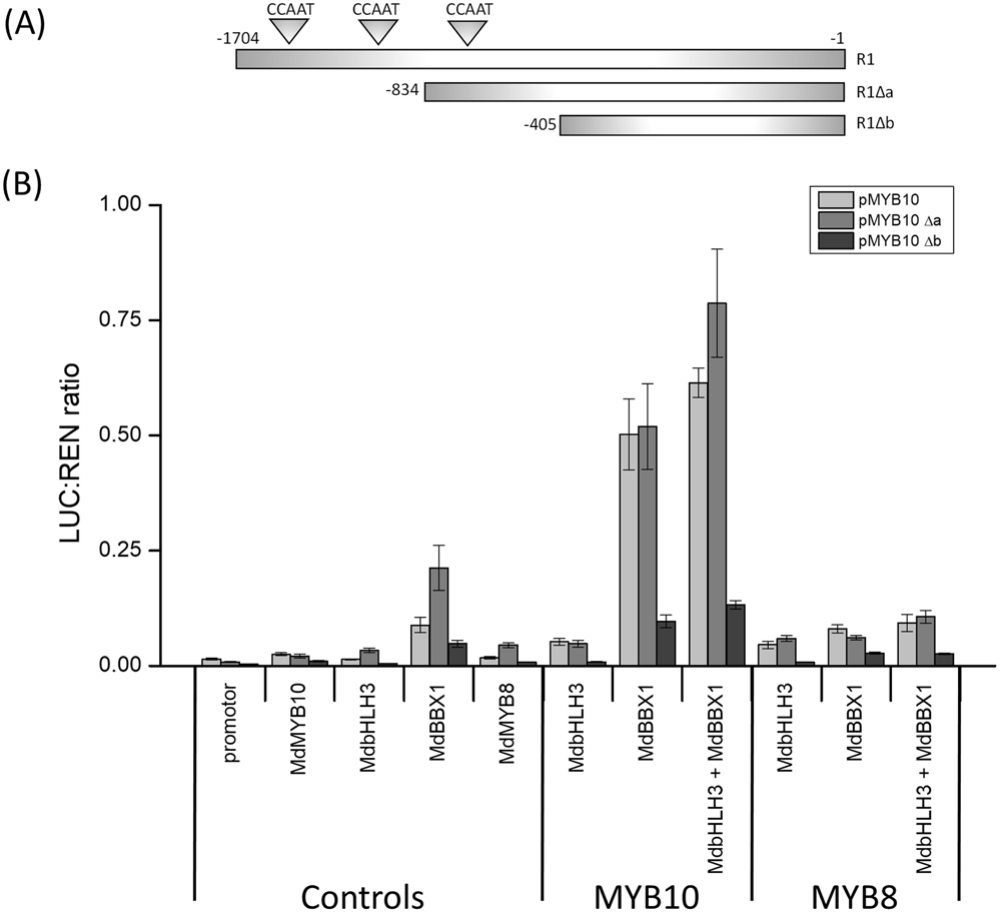
Identification of the region of the *MYB10* promoter activated by BBX1. (**A**) Schematic of the full length and two truncated versions (Δa and Δb) of the *MYB10* promoter used to identify the region sensitive to the presence of BBX1. Position of three CCAAT motifs indicated. (**B**) Transient expression assay using the full length *MYB10* promoter and the two truncated versions. Full length and truncated promoters were run alone, with MYB10, and with MYB8 to test activation capability of BBX1 with and without a bHLH cofactor. Error bars show SE of four biological replicates.

To further characterise the activation of the *MYB10* promoter by *BBX* genes, we generated two truncated versions of the 1704 bp promoter sequence of *MYB10* (Fig. 5A), *MYB10∆a* and *MYB10∆b* at 834 bp and 405 bp, respectively, upstream of the ATG start site. We assessed the promoter sequence for *cis*-elements which might facilitate interaction with BBXs and found CCAAT motifs present. In yeast these motifs are bound by HEME ACTIVATOR PROTEINS (HAP), also known as NUCLEAR FACTOR Y (NF-Y) proteins, facilitated by domains similar to the CCT domain of many plant *BBX*s in plants, including *BBX*^78,79^. Due to some *BBXs* containing the CCT domain it is possible they will bind the same CCAAT motif as the HAP proteins. The full *MYB10* promotor sequence contains three CCAAT motifs, *MYB10∆a* contains one, while *MYB10∆b* contains none. The infiltration of *BBX1* alone resulted in a slight activation of *MYB10* and *MYB10∆a* compared with MYB10 or bHLH3 controls (Fig. 5B). Most notably, the ability for the promoter to be activated by MYB10 and BBX1 dropped significantly for the *Δb* promotor sequence (Fig. 5B). This also applied if bHLH3 was present. Apple *MYB8* was included as a control to show specificity of the *MYB10* infiltration co-factor to *BBX1* activation of the promoter^22^.

### The apple DFR promoter is activated by BBX1

**Figure 6:**
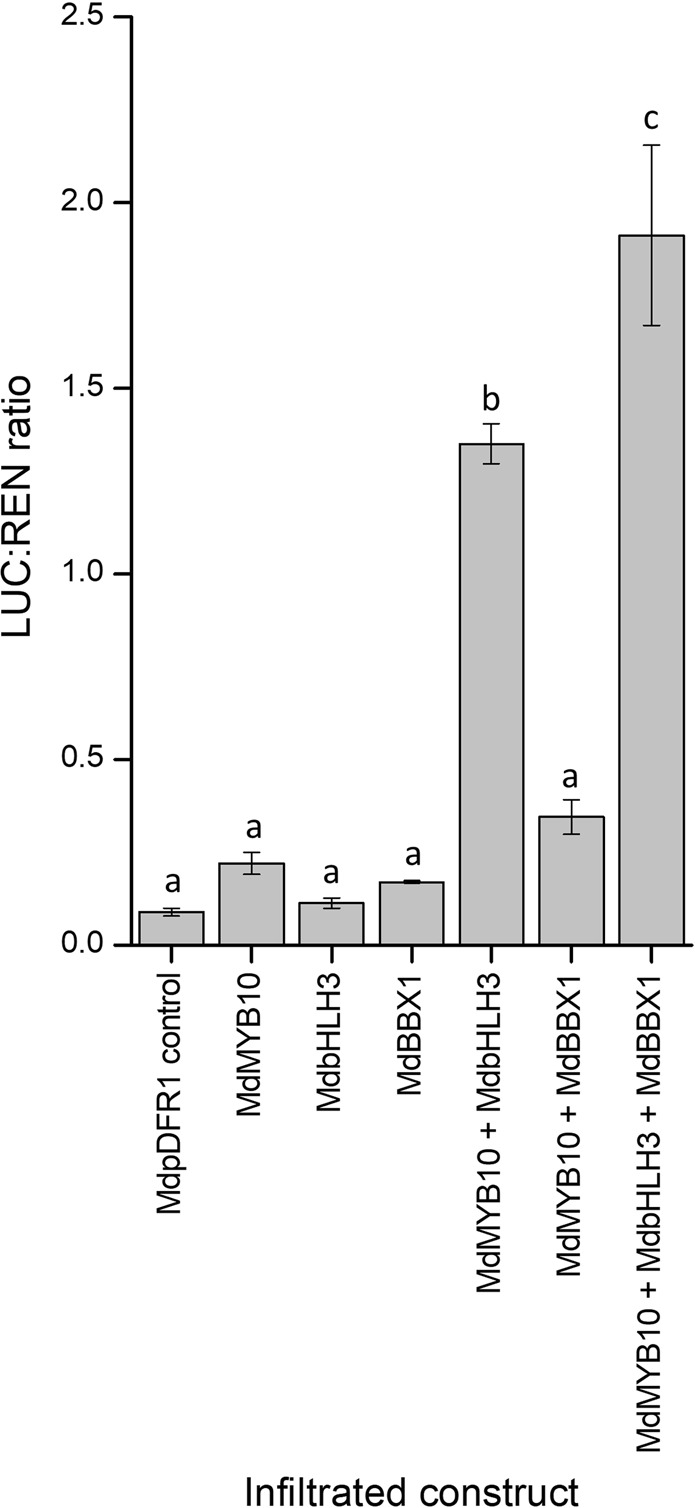
Transient expression assay of the activation of the promoter of the anthocyanin biosynthetic gene *DFR* by BBX1 in the presence of MYB10 and bHLH3. Error bars show SE of four technical replicates. Differences between averages were statistically analysed using ANOVA and pairwise comparison performed using Fisher’s LSD test at the 5% significance level. Error bars show SE of four biological replicates.

Having demonstrated that some BBXs activated the *MYB10* promoter (Fig. 4), the promoter of a gene encoding an anthocyanin biosynthetic enzyme, *DFR*, was selected to test for activation by BBX1. The promoter of *DFR* was selected as it displayed the most significant correlation with the expression of MYB10 (Fig. 3) and is a key step in the anthocyanin pathway, being one of the late biosynthetic enzymes. BBX1 was chosen because of its gene expression profile, both in the fruit development and diurnal rhythm experiments as well as its ability to activate the *MYB10* promoter. Infiltrations were performed in *N*. *benthamiana* using the dual luciferase assay (Fig. 6). Infiltration of *BBX1* alone did not activate the *DFR* promoter. Co-infiltration of both MYB10 and bHLH3 resulted in strong activation and this was further strengthened by the addition of BBX1. We checked the *DFR* promoter sequence for CCAAT motifs, finding two, one at −425 and the other at −659 upstream of the start site.

### Over-expression of BBX1 in transgenic ‘Royal Gala’ apple

**Figure 7:**
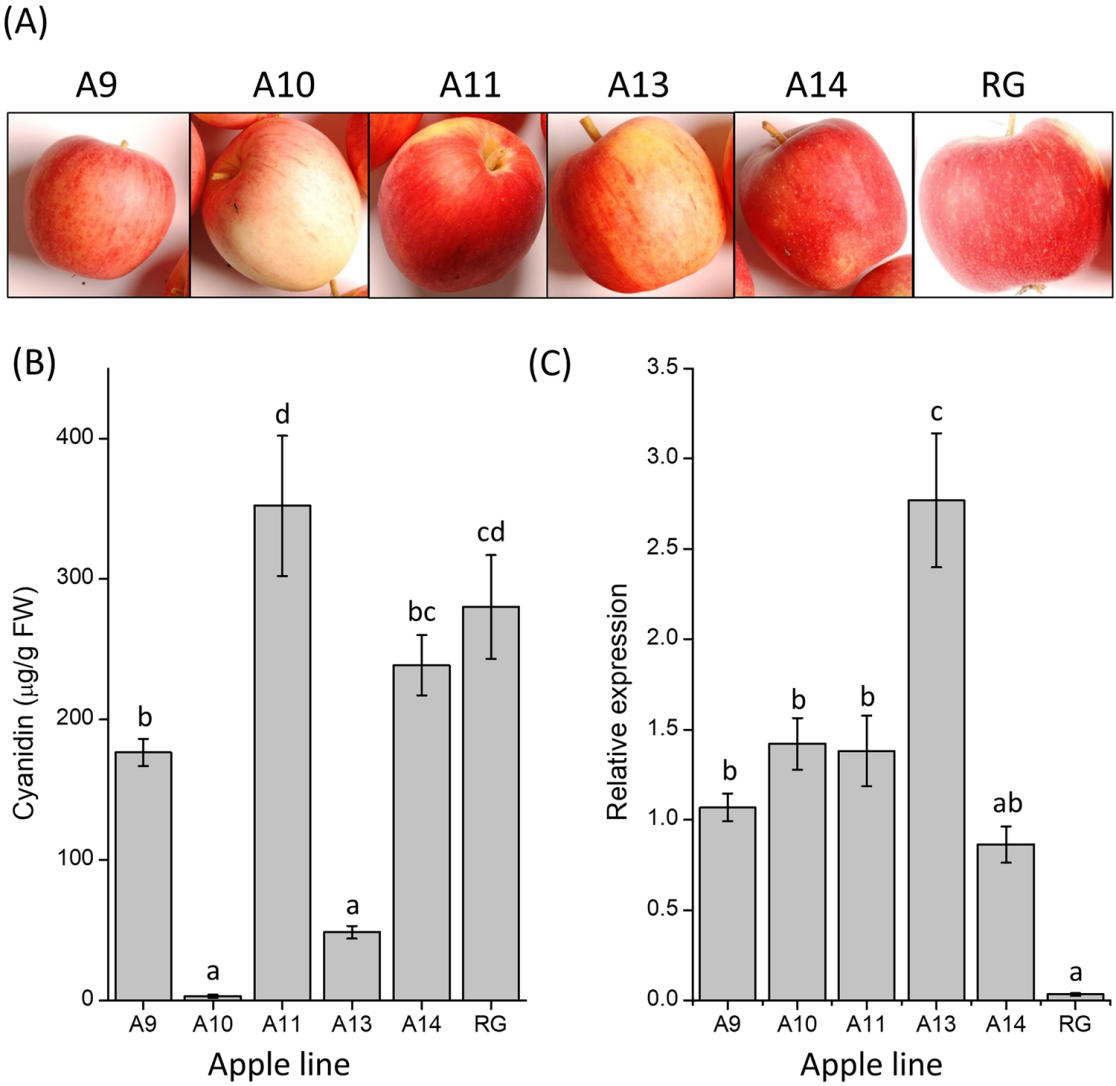
Characterisation of 35S:BBX1 transgenic lines. (**A**) Images of representative apples from each of the lines. (**B**) Cyanidin concentration in fruit skin for the transgenic lines compared with ‘Royal Gala’ control (RG). Cyanidin concentrations reported are the sums of cyanidin 3-*O*-galactoside, cyanidin 3-*O*-glucoside, and cyanidin 3-*O*-arabinoside in µg/g FW. Cyanidin 3-*O*-galactoside accounted for over 90% in every sample tested. (**C**) Gene expression of *BBX1* in the 35S:BBX1 transgenic lines compared with that in ‘Royal Gala’ control fruit (RG). Results are average expression of three biological replicates and four technical replicates relative to *Actin*. Error bars show SE of four biological replicates. Differences between averages were statistically analysed using ANOVA and pairwise comparison performed using Fisher’s LSD test at the 5% significance level.

The apple tree cultivar ‘Royal Gala’ was transformed with the *BBX1* cDNA driven by the CaMV35S promoter. Trees were grown in the glasshouse until sufficiently mature to flower. Apple fruit from five independent transgenic lines were harvested and phenotypically assessed 125 DAFB (Fig. 7A). HPLC analysis confirmed that the concentration of cyanidin-3-*O-*galctoside varied in the different lines, but mainly showed a reduction compared with the control (Fig. 7B). This suggests that other partners of this transcription factor are required for activation. The over-expression of *BBX1* in transgenic fruit was confirmed by RT-qPCR and was shown to be highly elevated in all the lines tested compared with the control (Fig. 7C). However, overexpression of BBX1 did not result in more anthocyanin accumulation in the fruit skin of transgenic apples. Fruit weight, firmness and soluble solids content, expressed in °Brix values were also recorded, showing a significant increase of firmness and soluble solids content in the over-expression lines compared with those in the ‘Royal Gala’ controls (Supplementary Fig. 4). Ethylene concentration and transcript abundance for *MYB10*, *DFR*, and ethylene pathway genes *ACS* and *ACS* were also assessed (Supplementary Fig. 5). Other polyphenolics in the over-expression lines were generally similar to those found in ‘Royal Gala’, although some differences were observed, such as an increase in chlorogenic acid (Supplementary Table 3).

## Discussion

In this study the putative role of *BBX* genes on anthocyanin accumulation in apple was assessed. This family of transcription factors control diverse processes in plants with at least one, BBX33, having a direct role in anthocyanin accumulation in apple fruit skin^7^. To study the *BBX* family we tested gene expression through fruit development and examined the possible role of a number of *BBX* genes in directly activating anthocyanin biosynthesis. Diurnal rhythms of candidate *BBX* genes were measured and *BBX1* was chosen to advance into stable transgenic work after showing the ability to activate *MYB10* and *DFR*.

### *BBX* transcriptional levels vary during fruit apple development

Anthocyanin accumulation in apple skin generally occurs late in fruit development and is a result of the anthocyanin-regulating MYB induction of the genes encoding anthocyanin biosynthetic enzymes. The expression patterns of a subset of *BBX* genes was assessed during fruit development and compared with both the anthocyanin content and the expression of *DFR*, *UFGT* and *MYB10*. This showed that *BBX* genes have a range of expression patterns, some of which increased with anthocyanin accumulation in fruit skin, similar to banana *BBX* expression during fruit development^80^. A number of these genes showed an expression patterns correlating with anthocyanin induction, including in particular *BBX1*, *15*, *33*, *51* and 54. *BBX1* showed the highest expression outside group IV *BBX*s. *BBX15*, *51* and 54 showed increasing expression during development, peaking during the period of highest anthocyanin accumulation, and these are all in group IV, which contains *BBX33*, already shown to activate the anthocyanin pathway in apple^7^. In Arabidopsis, group IV *BBX*s, in particular, have been identified as linked to light response and so were of interest to assess for any diurnal rhythms they might possess^81^.

### The expression pattern of *BBX* genes demonstrate a diurnal rhythm

BBX genes have been shown to be diurnally regulated in banana and potato^80,82^. In apple, expression analysis over a 48-hour period late in fruit development was analysed (Fig. 2). Strong patterns in expression were seen for *GI*, light, and temperature-related genes in fruit skin, and these daily rhythms appeared stable in the orchard-grown fruit. *BBX1*, *15*, *33* and 51 were chosen based on their expression patterns during fruit development. All four *BBXs* displayed diurnal patterns of expression for both seasons, except for *BBX33*, which displayed no obvious pattern for season 1. *BBX33* influences anthocyanin in apple^7^ and appears to have been influenced by an unknown factor in season 1 that did not affect *BBX1*, *BBX15* or *BBX51*. The diurnal variation in *BBX* transcription recorded here signifies the importance of sampling times for *BBX*s, and indeed all environmentally regulated genes, when considering experimental design. For example, results varied as much as six-fold for *BBX1* between sampling at 0800 h or 2000 h.

### The expression of anthocyanin-related genes demonstrates a clear diurnal rhythm, co-ordinated by MYB10

The diurnal rhythm demonstrated by a number of the *BBX* genes suggests that diurnal rhythms may also be important in anthocyanin regulation. We tested key genes in the anthocyanin pathway, and showed that transcript accumulation of these anthocyanin biosynthetic genes followed a very similar pattern over the 48-hour sampling period (Fig. 3). This was also evident for the MYB regulator, *MYB10*, confirmed by statistical analysis. This suggests that the pathway genes are modulated in a highly controlled manner, although further work is necessary to examine the interplay of the transcriptional control and environmental factors determining this phenomenon. We did not find any correlation between expression patterns of *MYB10* and *BBX1*, *15*, *33* or 51 (Supplementary Table 2A). Interestingly, *BBX1* did have a significant correlation with temperature (Supplementary Fig. 2B), a relationship supported by previous findings^7,32^. Temperature readings during the sampling period are provided in Supplementary Fig. 6. Furthermore, *BBX33* transcription had a significant correlation with *HY5* transcription for season 1, supporting the model that *BBX33* influences anthocyanin via *HY5* proposed by Bai *et al*.^7^ (Supplementary Table 2C). This correlation, however, was not seen for season 2 and so appears to vary seasonally and geographically.

### BBX genes can directly activate the apple anthocyanin-regulating MYB

To further determine the role of *BBX*s, we tested different sub-groups of the *BBX* family for transactivation of the promoter of the anthocyanin regulator MYB10 using luciferase assays. Activation capability was not specific to particular groups. As *BBX33* has been published by Bai *et al*.^7^ and group IV has been reported as important in light signalling^81^, we focused on this group by testing more of its members. There was substantial variation in the effect of addition of *MYB10* and *bHLH3*; for example, *BBX15* had little activation without *MYB10* and *bHLH3*, whereas *BBX17* had the same degree of activation regardless of the presence of *MYB10* or *bHLH3*. The highest activating *BBX*s were *BBX1*, 35, 15, 51 and 54, to which the closest predicted amino acid (AA) BLAST hits in Arabidopsis were AtBBX5 (AtCOL4), AtBBX12, AtBBX21, and AtBBX24, respectively (with apple BBX51 and 54 both being most similar to AtBBX24). In Arabidopsis these *BBX*s have been reported to be involved with photosynthesis, photomorphogenesis, and various stress responses^57,83^. AtBBX21 and AtBBX24 specifically have been linked to the regulation of *HY5*^84^, which is supported by results here that showed developmental transcription, diurnal rhythm, and *MYB10* activation for the apple homologues BBX15 and BBX51. Compared with the highest activating *BBX*s, *BBX33* did not highly activate *MYB10*, which, being involved in anthocyanin accumulation, suggests a low threshold is required for BBX activation of the anthocyanin pathway. This ability of *BBX*s to activate *MYB10* suggests the presence of *cis* elements in the *MYB10* promoter that might be bound by BBX proteins.

### A BBX-related *cis* element is necessary for BBX transactivation of the *MYB10* promoter

Data from the promoter deletion study (Fig. 5) showed that BBX activation of the *MYB10* promoter is partly dependent on the presence of upstream sequences. The shortest promoter fragment, ∆b, was not able to activate the reporter to the same extent as either the full promoter tested or ∆a. This suggests that BBX1 requires a motif that is absent in the most truncated version. Previous research on CONSTANS binding with the *FLOWERING LOCUS T* (*FT*) promoter has identified two possible motifs in Arabidopsis. A CCAAT box binding domain was identified by Wenkel *et al*.^78^, and this relatively common eukaryotic cis-element binds with the NF-Y/HAP complex to drive transcription. The full-length *MYB10* promoter showed a high level of transactivation in the presence of BBX1 (Fig. 5B). There are three CCAAT *cis*-elements in the 5’ region of this promoter sequence (Fig. 5A). There was no significant reduction when the same assay was performed using the truncated ∆a promoter fragment. This contains just one CCAAT domain. However, when ∆b containing no CCAAT motifs was used, there was a dramatic reduction in activation. This suggests the region between *MYB10∆a* and *MYB10∆b* is crucial for the effects of BBX1 and indicates that the CCAAT motif may be essential for BBX binding. However, there was still some activation, indicating that another element may be present. Bai *et al*.^7^ reported activation of *MYB10* also being influenced by ACGT-containing elements and Polymorphic G-boxes, and found activation occurring down to a −314 bp promotor fragment, suggesting a possible binding site between −405bp and −314bp. PLACE (Plant cis-acting regulatory DNA elements) analysis for this region identified many potential sites, but no CCAAT, ACGT-containing elements, or G-boxes. A second, more specific element has been described for CO binding of the *FT* promoter^85^. This sequence was identified as TGTG(N2-3)ATG, but was not found in the *MYB10* promoter. As well as the *MYB10* promotor, light-induced factors could also be upregulating the anthocyanin biosynthetic genes directly by binding their promotors.

### *BBX1* in combination with *MYB10* and *bHLH3* activates the DFR promotor

Previous results indicated that light responsive regulators of anthocyanin could be acting on the structural genes in the pathway directly^38,86^. Transient expression assays were performed on the promotor of *DFR* to test this for *BBX1*. *BBX1* did not display a significant activation of the *DFR* promotor when infiltrated alone (Fig. 6). As expected, a strong activation was seen when the *DFR* promoter was infiltrated with *MYB10* and *bHLH3*, which was enhanced with the co-infiltration of *BBX1*, suggesting some combinatorial effect of members of the MBW complex and BBX1. *BBX33* has previously been shown to be regulated by HY5 and to regulate MYB10^7^.

### *BBX1* overexpression in apple may affect fruit maturation, but did not increase anthocyanin accumulation

*BBX1* was over-expressed in apple trees. Fruit weight was not significantly different between the transgenic lines and the ‘Royal Gala’ control. There was, however, a significant difference in fruit firmness and soluble solids content (Supplementary Fig. 4). Ethylene was also significantly lower in the transgenic fruit (Supplementary Fig. 5A), which alongside the difference in firmness and soluble solids content, suggests an effect on fruit maturity. Transcript abundance for ethylene genes did not show the same pattern as ethylene measurements, which were all reduced in the over-expressing lines, suggesting the control of other factors (Supplementary Fig. 5B). *BBX*s have been linked to regulating shade avoidance, which is controlled by hormones that influence plant growth, such as ethylene^87^. The anthocyanin content of the transgenic fruit was either similar to control fruit, as shown in the independent lines A11 and A14, or reduced as shown in A9, A10, A13 (Fig. 6B). Although the expression of *BBX1* was high compared with that in the ‘Royal Gala’ control (Fig. 6C), the expression levels of the other genes tested were more similar to *MYB10* than to *BBX1* (Supplementary Fig. 5B), indicating that the over-expression did not overcome the control *MYB10* has over the anthocyanin pathway, particularly as the anthocyanin content correlated to the transcript abundance of *MYB10*. Other environmental conditions, such as temperature, are likely to contribute to the results of this study. It is possible that thresholds of temperature sensitive expression of *BBX1* were not reached during this work, and therefore anthocyanin accumulation was not affected. Future experiments could test this by manipulating temperature and measuring *BBX1* expression along with anthocyanin concentration.

## Conclusion

*BBX* genes play a diverse set of roles in plants. The few reports to date have classified the family and focused on individual *BBX*s^7,59^. Here, a wide range of *BBX*s were scrutinised to determine their potential as activators of the anthocyanin pathway, identifying candidates worth pursuing. The expression of a number of *BBX*s correlated with cyanidin-3-*O-*galactoside during fruit development. *BBX1* activated the promoters of *MYB10* and *DFR;* however, when *BBX1* was over-expressed in apple, there was no apparent increase in anthocyanin. Taken together, this evidence suggests that there may be an element of control of the anthocyanin pathway by *BBX*s with other partner transcription factors. Identifying transcriptional partners that influence colour in apple alongside the previously demonstrated effect of *BBX33*^7^ will aid the development of apple varieties with predictable colour development.

## Supplementary information

Supplementary Figures
