# Supplementary material for: Apple B-box factors regulate light-responsive anthocyanin biosynthesis genes
Source: Sci Rep. 2019 Nov 28;9:17762. doi: 10.1038/s41598-019-54166-2 (PMC6882830; doi:10.1038/s41598-019-54166-2)
Supplement: Supplementary file 1 — Supplementary Figures [file 41598_2019_54166_MOESM1_ESM.docx]

**Apple *B-box* factors regulate light-responsive anthocyanin biosynthesis genes**

Blue J. Plunkett, Rebecca Henry-Kirk, Adam Friend, Robert Diack, Susanne Helbig, Katriina Mouhu, Sumathi Tomes, Andrew P. Dare, Richard V. Espley, Joanna Putterill, Andrew C. Allan.

**Supplementary Figure and Table legends**

Table S1: Primers used for qPCR.

Figure S1: Relative expression of *BBX* genes during fruit development. Samples were taken at seven time points during development: 35, 65, 85, 110, 120, 130, and 140 DAFB. All sampling occurred at 1300 h. Error bars show SE of four technical replicates.

Figure S2: Relative expression of additional genes tested in ‘Royal Gala’ apple leaves from season 2 in Auckland.

Figure S3: Additional temperature and clock associated genes tested in fruit skin for two seasons at seven time points during development. All sampling occurred at 1300 h. Error bars show SE of four technical replicates.

Table S2: Correlation analysis tables for relationship between; (A) *BBX* genes with *MYB10,* (B) *BBX* genes and *MYB10* with temperature, (C) *BBX* genes and *MYB10* with *HY5* (*p*-value = <0.05).

Figure S4: Fruit weight (g), firmness (kgf), and soluble solids content (°Brix) of *BBX1* over-expressing lines compared with ‘Royal Gala’ controls. Values are the mean of five fruit and error bars show SE. Differences between averages were statistically analysed using ANOVA and pairwise comparison performed using Fisher’s LSD test at the 5% significance level.

Figure S5: Ethylene and gene expression data for over-expressing lines and ‘Royal Gala’ controls. (A) Average internal ethylene concentrations of transgenic apple lines expressing *BBX1* driven by the 35S promoter compared with those in control ‘Royal Gala’ (RG) fruit. Values are the mean of readings from five fruit. (B) Gene expression of *MYB10, DFR, ACS3,* and *ACO1* in the fruit skin of *35S:BBX1* transgenic lines. Values are average expression of three biological replicates and four technical replicates relative to *Actin*. Error bars show SE. Differences between averages were statistically analysed using ANOVA and pairwise comparison performed using Fisher’s LSD test at the 5% significance level.

Table S3: Metabolite analysis for 35S:BBX1 transgenic lines. Data are results for three replicates and a pool of the three for each of the over-expressing lines and ‘Royal Gala’ (RG) controls, presented as ug/g FW.

Figure S6:

Apple skin temperature during diurnal experiments. Season 1 in Nelson (left) and season 2 in Auckland (right). Samples are mean temperature readings from six channel data loggers placed under on-tree ‘Royal Gala’ fruit skin in the orchard.


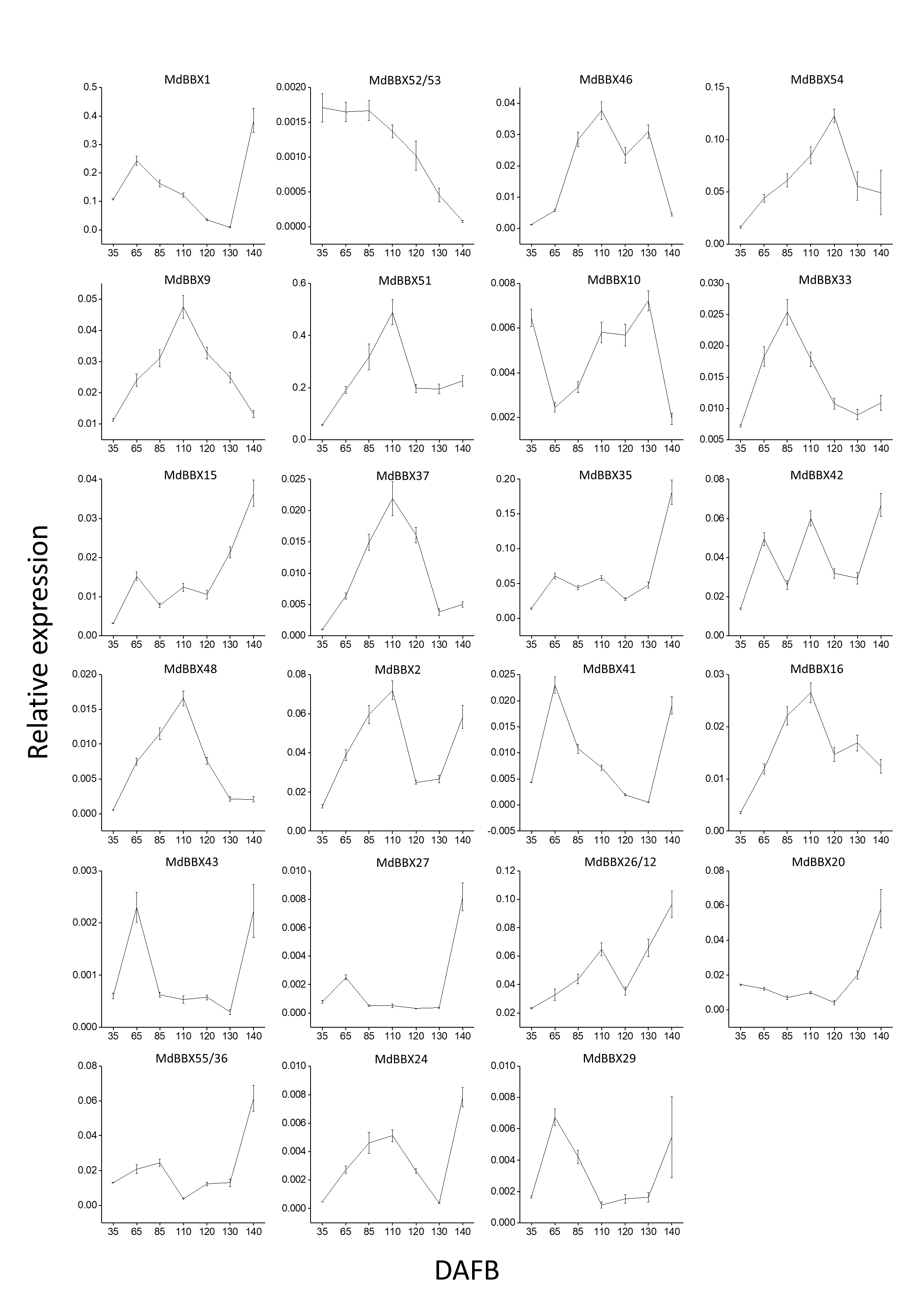


Supplementary Figure 1


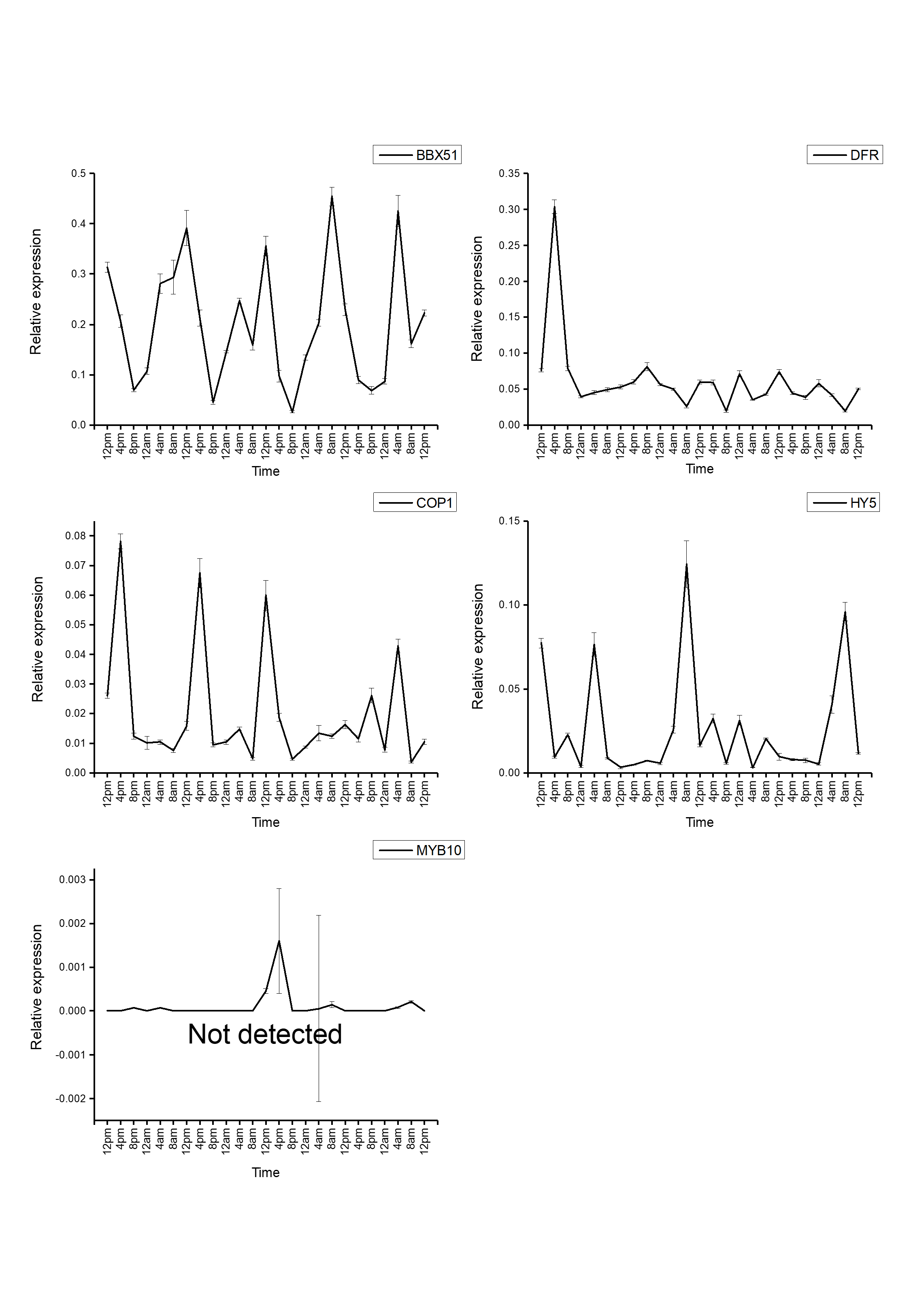

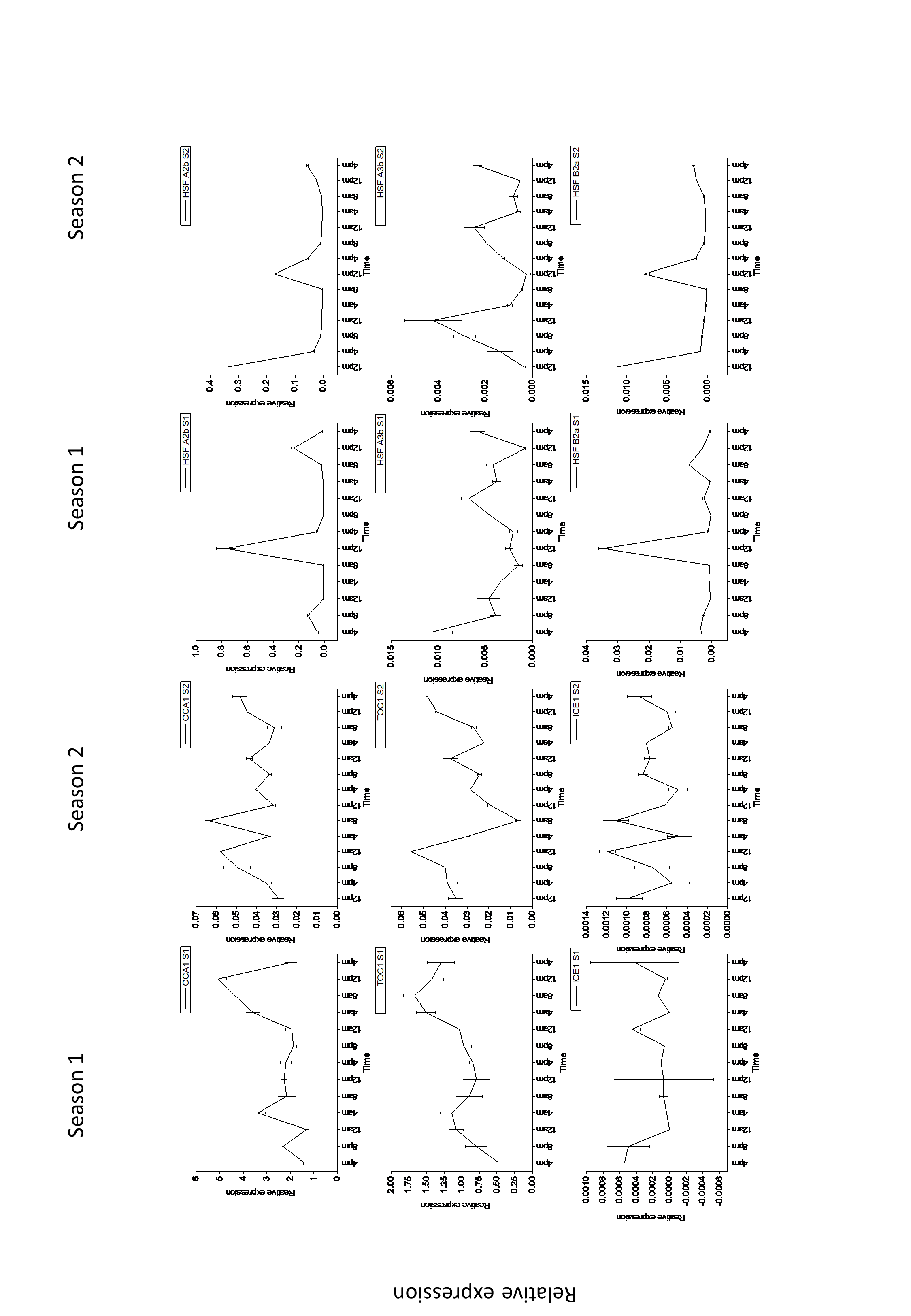

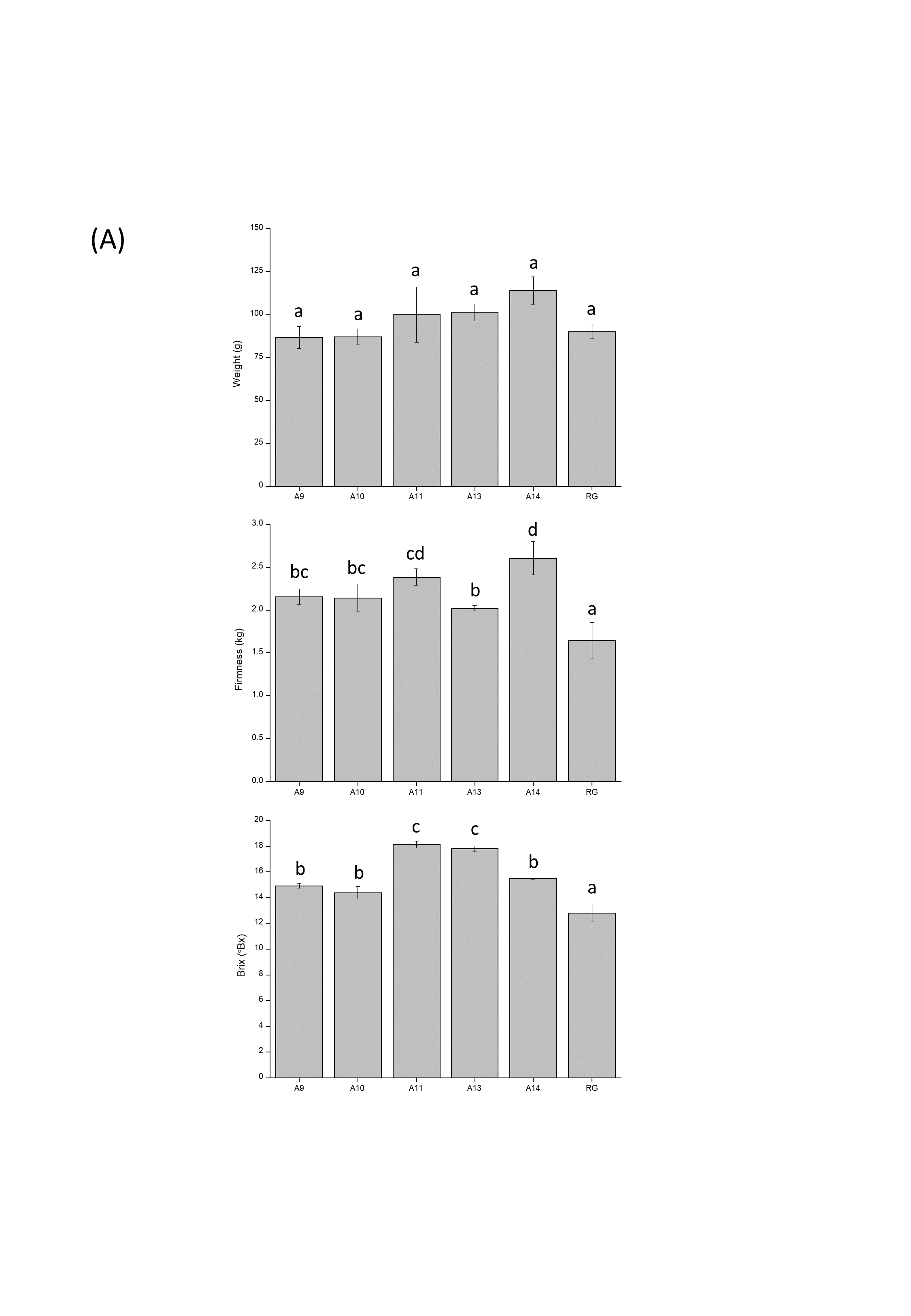

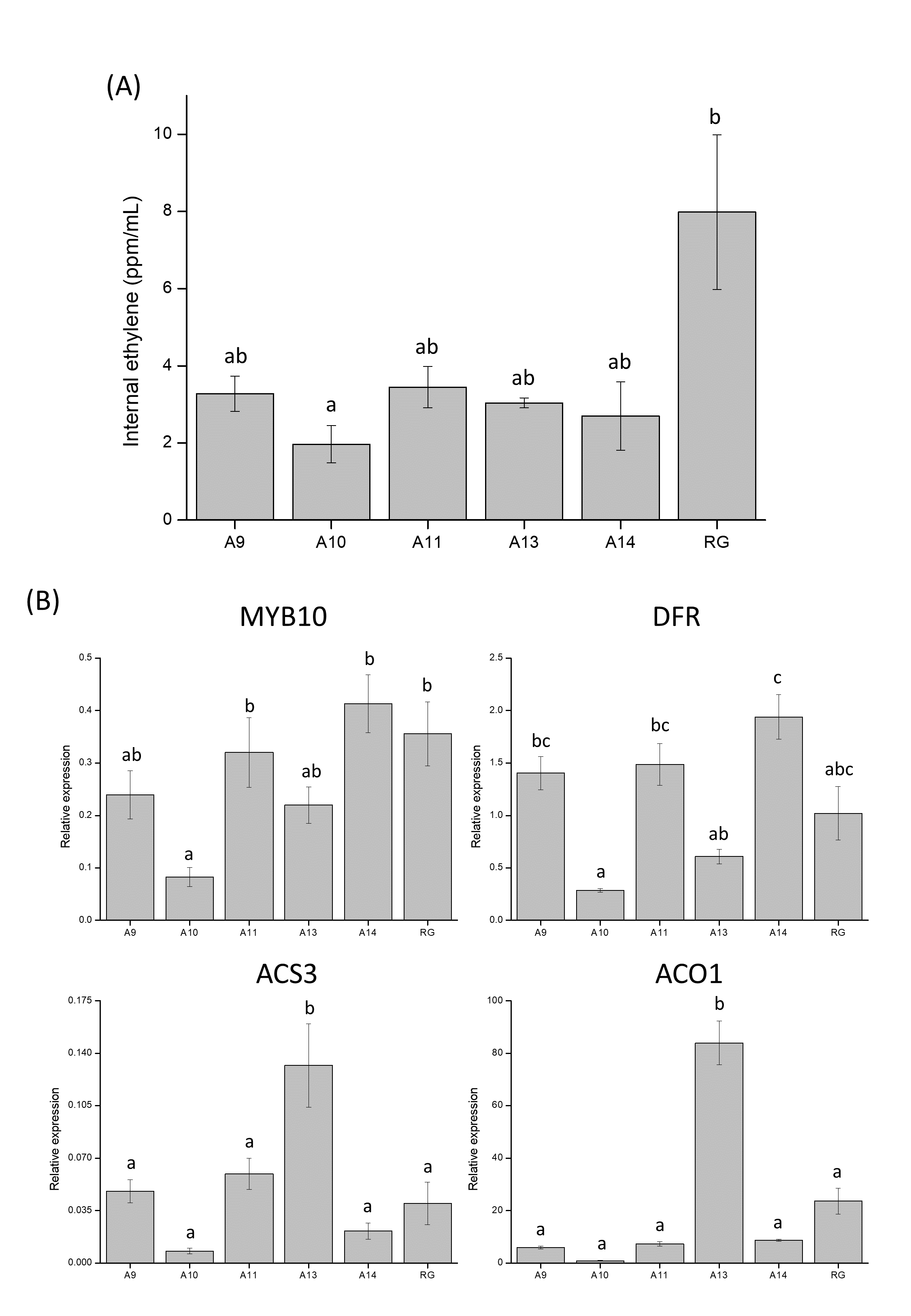

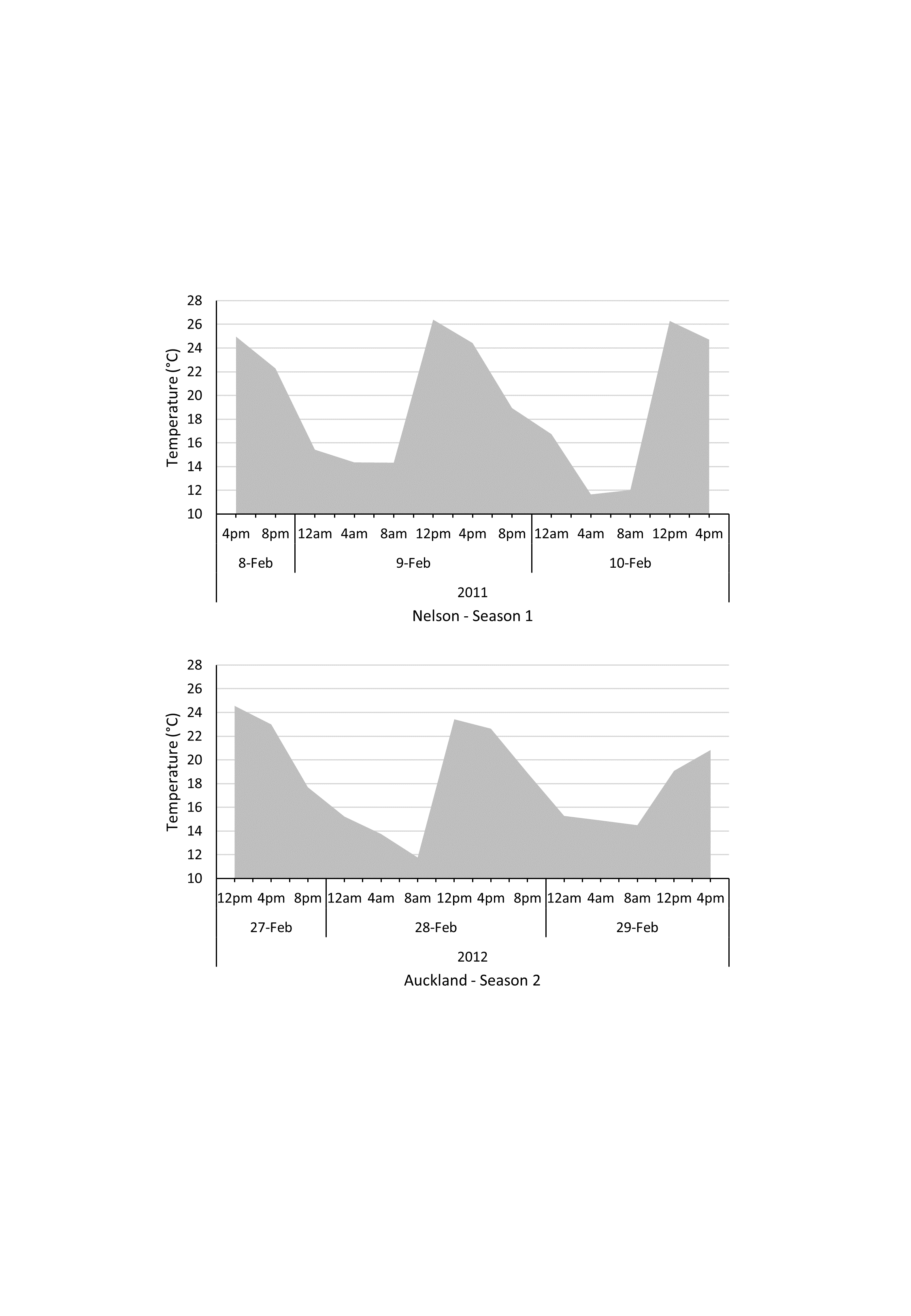


Supplementary Figure 2

Supplementary Figure 3

Supplementary Figure 4

Supplementary Figure 5

Supplementary Figure 6
